# Disease trajectories and mortality among women diagnosed with breast cancer

**DOI:** 10.1186/s13058-019-1181-5

**Published:** 2019-08-16

**Authors:** Haomin Yang, Yudi Pawitan, Wei He, Louise Eriksson, Natalie Holowko, Per Hall, Kamila Czene

**Affiliations:** 1 Department of Medical Epidemiology and Biostatistics, Karolinska Institutet, Nobels Väg 12A, 171 77 Stockholm, Sweden; 2 Department of Oncology Pathology, Karolinska Institutet, Stockholm, Sweden

**Keywords:** Breast cancer, Disease trajectory, Mortality

## Abstract

**Purpose:**

Breast cancer is a common disease with a relatively good prognosis. Therefore, understanding the spectrum of diseases and mortality among breast cancer patients is important, though currently incomplete. We systematically examined the incidence and mortality of all diseases following a breast cancer diagnosis, as well as the sequential association of disease occurrences (trajectories).

**Methods:**

In this national cohort study, 57,501 breast cancer patients (2001–2011) were compared to 564,703 matched women from the general Swedish population and followed until 2012. The matching criteria included year of birth, county of residence, and socioeconomic status. Based on information from the Swedish Patient and Cause of Death Registries, hazard ratios (HR) were estimated for disease incidence and mortality. Conditional logistic regression models were used to identify disease trajectories among breast cancer patients.

**Results:**

Among 225 diseases, 45 had HRs > 1.5 and *p* < 0.0002 when comparing breast cancer patients with the general population. Diseases with highest HRs included lymphedema, radiodermatitis, and neutropenia, which are side effects of surgery, radiotherapy, and chemotherapy. Other than breast cancer, the only significantly increased cause of death was other solid cancers (HR = 1.16, 95% CI = 1.08–1.24). Two main groups of disease trajectories were identified, which suggest menopausal disorders as indicators for other solid cancers, and both neutropenia and dorsalgia as diseases and symptoms preceding death due to breast cancer.

**Conclusions:**

While an increased incidence of other diseases was found among breast cancer patients, increased mortality was only due to other solid cancers. Preventing death due to breast cancer should be a priority to prolong life in breast cancer patients, but closer surveillance of other solid cancers is also needed.

**Electronic supplementary material:**

The online version of this article (10.1186/s13058-019-1181-5) contains supplementary material, which is available to authorized users.

## Introduction

Breast cancer is the most common cancer diagnosed among women worldwide, with 80–90% of patients surviving more than 5 years after diagnosis [1]. Breast cancer patients have an increased risk of recurrences, early side effects, and with increasing survival, late adverse health effects related to both the disease and therapy. Previous studies on diseases associated with breast cancer have focused on a limited number of diseases [2–4]. Available evidence regarding the incidence of, and mortality due to, other diseases after breast cancer diagnosis is limited by small sample sizes, limited number of diseases selected, and/or short-term follow-up [5–8]. As a result, the global spectrum of diseases and mortality among breast cancer patients has not been thoroughly addressed.

Since the effectiveness of clinical care for breast cancer patients depends on early detection and intervention for adverse outcomes, analyzing sequential patterns of disease occurrence (disease trajectories) may help identify key diagnoses to mitigate the risk of future poor outcomes. While disease trajectories are commonly identified in the general population [9, 10], to date no study has been conducted among breast cancer patients, specifically studying the sequential associations between early treatment-related side effects and later life-threatening outcomes.

This study comprehensively investigated disease progression by (i) assessing the risk of disease incidence and mortality among breast cancer patients, compared to the general population, and (ii) analyzing disease trajectories in breast cancer patients to identify targets for early preventive interventions.

## Materials and methods

A flow chart illustrating the study populations for analyses is found in Additional file 1: Figure S1.

### Study populations

A Swedish national cohort of breast cancer patients was identified through the Swedish Cancer Registry, which was founded in 1958 and has almost 100% coverage of cancer patients in Sweden [11]. The inclusion criteria were age at diagnosis (20–80 years), year of diagnosis (2001–2011), and being part of the 1990 Swedish national census, resulting in 57,501 patients. The upper limit of age 80 years was set considering the relatively poor quality of cause of death information in the registry for women beyond this age. Disease diagnoses other than cancer were obtained from the primary and contributory diagnoses recorded in the Swedish Patient Registry [12], which was established in 1964 and has national coverage for inpatient hospitalizations since 1987. From 2001, this registry also includes hospital-based outpatient physician visits. Other cancer diagnoses were also obtained from Swedish Cancer Registry, while vital status and cause of death was retrieved from the Swedish Cause of Death Registry. The Swedish Cause of Death Registry has more than 99% completeness for information on underlying cause of death [13], with the validity varying between 80 and 90% among those who died before age 80 years [14].

Diagnoses and cause of death were coded according to the Swedish revision of the International Classification of Diseases (ICD) codes. The ICD-10 system (used since 1997) has a clear hierarchical structure for disease classification. All diseases in this study were defined based on the 3-digit ICD-10 codes (A00 to N99). We additionally combined several ICD codes, considering their biological and clinical similarity (e.g., combining F40 and F41 for anxiety) (Additional file 2). Only diseases with more than 100 cases among the cohort of breast cancer patients were included for analysis. The same range of diseases were used for the analysis of underlying cause of mortality.

### Disease diagnoses and mortality

To compare the incidence and mortality of other diseases in breast cancer patients, we randomly sampled up to 10 women from the Swedish population as our control group (Additional file 1: Figure S1). Matching was selected based on year of birth, county of residence, and socioeconomic status (obtained from the 1990 Swedish national census, and categorized as blue collar workers, white collar workers, self-employed workers, farmers, and others). Each control was alive and free of breast cancer 2 months after the matched patient’s cancer diagnosis (the index date), resulting in 564,703 matched controls (Table 1). Follow-up of both breast cancer patients and matched individuals started from the index date and ended on December 31, 2012 (December 31, 2011, to estimate the incidence of other cancers), date of death, date of emigration, or date of the studied disease diagnosis (or mortality), whichever came first. Information on death and emigration was obtained by using the unique Swedish personal identification numbers to cross-link cohorts with the Swedish Cause of Death Registry and the Swedish Migration Registry. Individuals with a previous disease diagnosis before the index date were excluded when analyzing that specific disease.

**Table 1:** Descriptive characteristics of breast cancer patients and their matched individuals (*N* = 622,204)

	Breast cancer patients *n* = 57,501	Matched individuals *n* = 564,703
Cohort period	2001–2012	2001–2012
Age at diagnosis (years)		
Mean (SD)	60.1 (11.0)	60.0 (11.0)
Min–max	20–80	20–80
Duration of follow-up (years)		
Median (IQR)	5.3 (5.5)	5.8 (5.5)
Total no. of person years at risk	320,742	3,340,912

Abbreviations: *SD* standard deviation, *IQR* interquartile range. The Swedish national cohort of breast cancer patients includes women diagnosed with primary invasive breast cancer between 2001 and 2011. In this cohort, follow-up is complete until December 31, 2012. Individuals from the general population are matched on year of birth, county of residence, and socioeconomic status (obtained from the 1990 national census of Sweden, categorized as blue collar workers, white collar workers, self-employed workers, farmers, and others)

### Statistical analysis

#### Disease incidence and mortality

In order to assess the disease incidence and mortality among breast cancer patients compared to healthy individuals, a matched-cohort analysis was performed using stratified Cox regression models, treating breast cancer as the exposure and disease incidence or mortality as the outcome. In total, there were 225 outcomes, with a sub-cohort formed to study each outcome. Based on Bonferroni correction to account for multiple testing, the threshold of significant *p* values was set to 0.00022.

#### Disease trajectory analysis

Disease trajectories after breast cancer diagnosis were studied by investigating the risk of one disease (D2, disease outcome) following the occurrence of another disease (D1, disease exposure) among the breast cancer patient cohort (Additional file 1: Figure S2). In the first step, in order to study disease trajectories independent of aging in the general population, only those diseases with significantly increased risk after breast cancer diagnosis were included. We also restricted analyses to disease pairs (D1 → D2) with more than 50 cases of D2 after D1 diagnosis.

As recommended by Jensen et al. [9], a disease pair can be identified as a disease trajectory if they occur in a sequential pattern and are significantly associated. Therefore, in the second step, we used a binomial test to assess the sequential pattern of D1 → D2, which is to test whether the probability of D2 being diagnosed after D1 was significantly larger than 50% among patients diagnosed with both D1 and D2. In the third step, for those pairs with a significant D1 → D2 sequential pattern, a nested case-control design was used to assess the association between D1 and D2, using D1 as time-dependent exposure and subsequent D2 as outcome. Follow-up for D2 started 2 months after breast cancer diagnosis. Since we are only interested in incident diseases after breast cancer, patients with either D1 or D2 before the start of follow-up were excluded. We defined breast cancer patients with D2 as cases and, using incidence density sampling, matched them with up to 5 breast cancer patients as controls. Each control was alive and free from D2 at the same follow-up time when D2 occurred in their matched case. In addition to the matching criteria used in the disease incidence analysis, we also matched patients for year of cancer diagnosis to account for calendar period effects. Conditional logistic regression models were used to calculate the odds ratio (OR) of D2 after D1, and significantly associated disease pairs with OR > 1 were considered for further combination into disease trajectories. OR from conditional logistic regression model with incidence density sampled data can be interpreted as HR in the cohort study.

In the fourth step to study disease trajectories leading to mortality, we further restricted the analysis for disease trajectories that were experienced by more than 20 patients (i.e., more than 20 breast cancer patients in the cohort had been diagnosed with D1 and thereafter D2, and consequently died). D2s in the trajectories were further tested for association with disease causes that were associated with increased mortality among breast cancer patients. This analysis was performed in the same manner as the D1 → D2 association analysis with a nested case control design. Significant trajectories were mapped separately, according to the cause of mortality.

To account for the multiple testing problem, the thresholds of significant *p* values were adjusted using Bonferroni correction.

Statistical analyses were performed using SAS (version 9.4; SAS Institute Inc., Cary, NC, USA), Stata (version 15.1; Stata Corporation, College Station, TX, USA), and R software (version 3.4.1; R Foundation for Statistical Computing, Vienna, Austria). The study was approved by the Regional Ethical Review Board in Stockholm.

## Results

### Incidence and mortality of other diseases in breast cancer patients, when compared with matched individuals

**Fig. 1:**
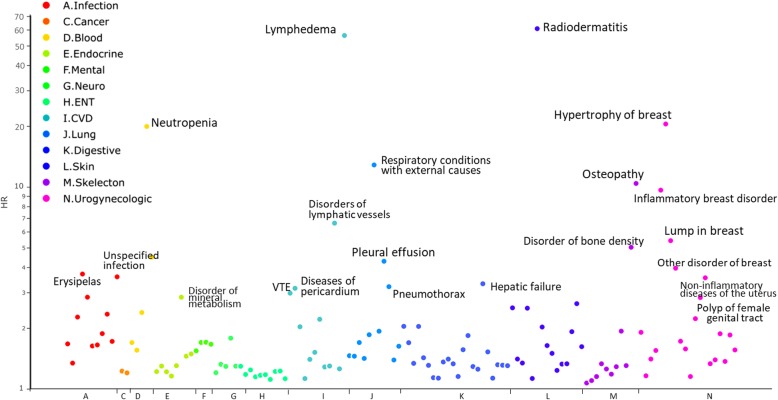
Significant hazard ratios (HRs) of diseases among breast cancer patients, compared to matched individuals (*N* = 622,204). All risk increases are statistically significant after considering the issue of multiple testing (*p* < 0.00022). The *Y*-axis shows the hazard ratio (on the log scale) of the disease in breast cancer patients, compared to healthy women who were matched on year of birth, county of residence, and socioeconomic status. The *X*-axis shows the disease categories according to ICD codes A-N. For example, lymphedema is classified under the disease category “circulatory system disease” (ICD-10 code I97). Breast cancer patients had a 56-fold increased risk of lymphedema, compared to matched healthy women. Details of the number of cases, hazard ratios, and confidence intervals are listed in Additional file 1: Table S1

Among the 225 diseases analyzed in the cohort, 45 diseases had an increased risk after breast cancer diagnosis with > 300 cases, HRs > 1.5, and *p* < 0.05/225(=0.00022) (Additional file 1: Table S1, Fig. 1). Diseases with the highest HRs included lymphedema, radiodermatitis, and neutropenia, all which are side effects of treatments corresponding to surgery, radiotherapy, and chemotherapy (Fig. 1). We also had some novel findings for diseases among breast cancer patients, such as the risk of larynx diseases (HR = 1.86, 95% CI = 1.67–2.07) and several gynecological disorders (Additional file 1: Table S1).

The most prevalent diseases among breast cancer patients (> 10%) had a HR close to one, including hypertension and disorders of the crystalline lens. One exception was a 56% (95%CI = 1.52–1.61) increased risk of menopausal disorders, representing a side effect of hormone therapy.

Compared to matched individuals, other causes of mortality were increased by 6% among breast cancer patients (Table 2). This increased mortality was mainly attributed to other solid cancers (HR = 1.16, 95% CI = 1.08–1.24). All other causes of mortality were not increased among breast cancer patients.

**Table 2:** Underlying causes of mortality among breast cancer patients. Hazards ratios (HR) with 95% confidence intervals presented (*N* = 622,204)

Disease	Code	No.	HR (95%CI)
Breast cancer mortality	C50	5405	**–**
Causes other than breast cancer		3556	*1.06 (1.02–1.10)*
Infectious diseases			
Overall		68	1.08 (0.84–1.39)
Sepsis	A41	35	1.19 (0.84–1.70)
Other cancers			
Overall		1314	1.14 (1.07–1.21)*
Other solid cancers	C00	890	1.16 (1.08–1.24)*
Hematological cancers	C81	76	1.11 (0.87–1.41)
Cardiovascular diseases			
Overall		1069	1.01 (0.94–1.07)
Hypertensive disorders	I10	27	1.00 (0.67–1.51)
Venous thromboembolism	I26	31	1.45 (0.99–2.13)
Cardiac arrhythmia	I49	38	1.11 (0.79–1.57)
Heart failure	I50	65	0.98 (0.76–1.28)
Stroke	I60	180	0.94 (0.80–1.09)
Other non-communicable diseases			
Overall		839	0.96 (0.89–1.03)

Hazard ratios of mortality causes among women in the Swedish national cohort of breast cancer patients, compared to women from the general population who were matched on year of birth, county of residence, and socioeconomic status. The table shows the most prevalent diseases among breast cancer patients. Results with *p* value < 0.05 are in italics

*Significant result after Bonferroni correction (*p* < 0.00022)

### Disease trajectories in breast cancer patients

**Fig. 2:**
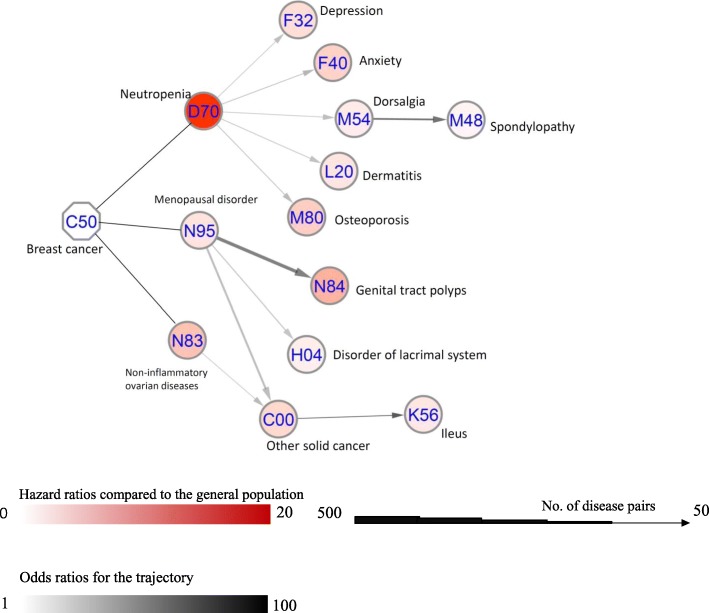
Overall trajectories of other diseases among breast cancer patients. This figure illustrates an overview of disease trajectories identified in our analysis. The combined ICD-10 codes for the diseases are shown within the circle. The color of the circle represents the hazard ratio of this disease among the breast cancer patients, compared to matched individuals. The width of the arrow connecting two circles corresponds to the number of breast cancer patients with this disease trajectory. The color of the arrows indicates the odds ratio of the sequential association between the two diseases. The strongest association in this figure is C00 → K56, suggesting a 15 times increased risk of ileus after other cancer diagnosis, with 83 patients in the cohort experiencing this trajectory. Trajectories starting with M15 and M20 were not included, given their low HR (HR ≤ 1.1) and that they were probably the result of surveillance bias

**Fig. 3:**
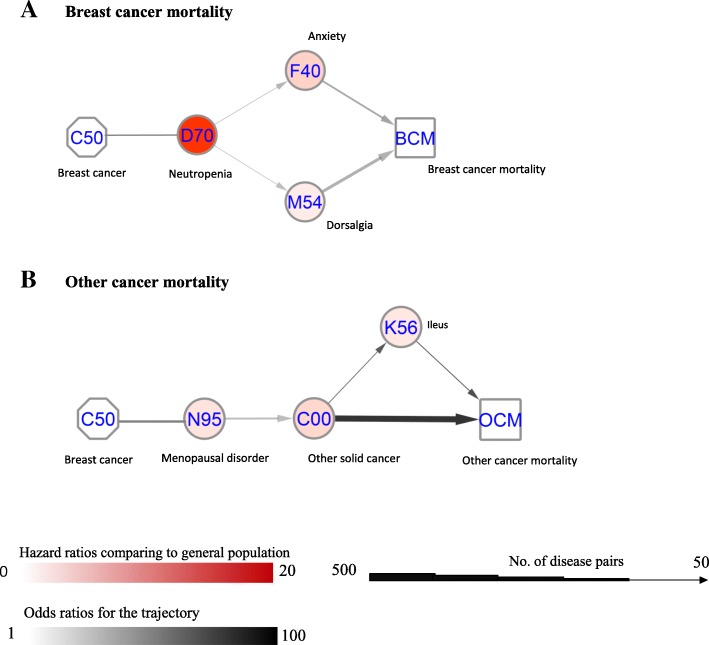
Disease trajectories leading to mortality among breast cancer patients. This figure shows the identified disease trajectories leading to breast cancer and other cancer mortality in our cohort. For each pair of the trajectory, the codes in the circle are the combined ICD-10 codes for the diseases. The color in the circle represents the hazard ratio of this disease among the breast cancer patients, compared to the matched individuals. The squares of **a** BCM and **b** OCM are breast cancer mortality and other cancer mortality. The width of the arrows between two circles (or square) corresponds with the number of breast cancer patients who had been first diagnosed with one disease and thereafter another. The color of the arrows indicates the odds ratio of the sequential association between the two diseases (or disease to mortality). In this figure, other solid cancer was associated with 72 times increased risk of other cancer mortality and 63 patients in the cohort had experienced the trajectory from menopausal disorder to other cancer mortality (N95 → C00 → OCM)

Altogether, 15 disease pairs were found to have an increased risk of D2 after D1 among breast cancer patients (*p* < 0.0013, Additional file 1: Table S2). Many of the disease trajectories were related to side effects of chemotherapy and hormone therapy, such as neutropenia, anxiety, osteoporosis, and menopausal disorders (Fig. 2). Since only the risk of breast cancer mortality and other cancer mortality were increased, when compared with the general population, the disease pairs were grouped into trajectories for each of these outcomes, where there were more than 20 cases (Fig. 3). Breast cancer mortality was associated with a previous diagnosis of neutropenia, dorsalgia, and anxiety (Fig. 3a), while menopausal disorders were associated with the incidence of other solid cancers and consequently other cancer mortality (Fig. 3b).

## Discussion

A systemic attempt to elucidate the risk profile for all other diseases after breast cancer diagnosis has previously not been performed. In this study, we found increased incidence of many infectious and non-communicable diseases after breast cancer diagnosis (see Fig. 1), confirming previous findings [2, 15–17]. We also identified several diseases of previously unknown risk among breast cancer patients, such as larynx diseases and several gynecological disorders. Since larynx disorders are associated with external hormones (e.g., oral contraceptives) [18], it is possible that this increased risk is a result of hormone therapy in breast cancer patients. Another novel finding was an increased risk of several non-inflammatory gynecological disorders, such as hyperplasia, cysts, and polyps of the female genital tract (Additional file 1: Table S1). Since tamoxifen has a known influence on the female genital tract [19], it is therefore not surprising to find an increased risk of such gynecological disorders among breast cancer patients.

Despite the increased risk of many diseases, we observed increased mortality only for other cancers (Table 2), suggesting that the lethality of diseases diagnosed among breast cancer patients was the same as in the general population. The increased incidence of and mortality due to other cancers among breast cancer patients has been reported previously with similar estimates [20, 21]. Since younger age at diagnosis and family history of breast cancer are both associated with an increased risk of other cancers after breast cancer diagnosis [22], shared genetic factors among different types of cancers might contribute to this increased risk. Moreover, treatments for breast cancer may also be carcinogenic and increase the risk of other cancers; the risk of lung cancer is increased after radiotherapy for breast cancer [23], while hormonal treatments may increase the risk of endometrial cancer [24].

The disease trajectory analysis in our study indicated several sequential associations for side effects of chemotherapy and hormone therapy among breast cancer patients (see Fig. 2). The risk of depression, anxiety, dermatitis, and osteoporosis was associated with a diagnosis of neutropenia, with all of these diseases being toxic side effects of chemotherapy [25–28]. These sequential associations also suggest that neutropenia is an early and instant sign for chemotherapy toxicity among breast cancer patients. It is therefore necessary to provide targeted interventions for this patient group (such as granulocyte colony-stimulating factors), in order to reduce side effects and promote treatment adherence. Other diseases identified in the overall trajectories are mainly associated with tamoxifen use, considering its effect on ovarian cysts and endometrial tumorigenesis [24, 29, 30].

Among the trajectories leading to breast cancer mortality, a diagnosis of neutropenia was also associated with increased mortality due to breast cancer (see Fig. 3a). Since neutropenia is a side effect of chemotherapy, and chemotherapy is usually given to patients with advanced tumors, it is reasonable to believe that neutropenia is associated with breast cancer mortality. We also found that dorsalgia and anxiety were associated with breast cancer mortality. Dorsalgia is long identified as a sign of bone metastasis [31], while the risk of anxiety is increased after chemotherapy and is associated with advanced tumor characteristics [27]. Indeed, all these three diseases/disorders are indicators of aggressive breast cancer tumor characteristics and consequently are associated with a worse disease prognosis. Our finding also suggests that clinicians (particularly general practitioners) and patients should pay special attention to dorsalgia symptoms, considering it is associated with bone metastasis.

In the disease trajectories leading to other cancer mortality, we found an increased incidence of menopausal disorders among breast cancer patients; these disorders are associated with the incidence of other cancers, ileus, and finally other cancer mortality (see Fig. 3b). Menopausal disorders are common side effects of hormone therapy, with a prevalence of more than 10% among breast cancer patients in the studied cohort. Symptoms in menopausal disorders such as uterine bleeding are established predictors for endometrial cancer [24], while ileus is a complication after gynecological surgery [32]. Since the risk of endometrial cancers is increased after tamoxifen treatment [33], but not aromatase inhibitors [34], a genital tract examination should be recommended during follow-ups for breast cancer patients being treated with tamoxifen.

There are several key strengths of our study. The large sample size of breast cancer patients and matched individuals retrieved from the Swedish national health registries provided a unique opportunity to detect a comprehensive spectrum of diseases with increased risk among patients in the real world, with an accurate estimation of HRs. The use of national registries also minimizes selection and information bias. In addition, analysis of disease trajectories allowed us to reveal key diagnostic indicators for poor survival.

However, we also acknowledge several limitations of this study. Although validity of the ICD codes used for disease diagnoses in the Swedish Inpatient Registry is about 85–95% [12], diagnoses from the outpatient registry have not as yet been validated. This could possibly result in disease misclassification, particularly for those diseases with slight to moderate symptoms and those defined by contributory diagnostic codes. Such misclassification should not have influenced our main findings, since the majority of our results relate to life-threatening diseases among breast cancer patients, and sensitivity analyses focusing on the main diagnosis did not differ greatly from the presented results (Additional file 1: Table S3, 80% of the significant point estimates had a difference of less than 10%). We were also limited by using the patient registry, which does not include diseases diagnosed and treated in a primary care setting and therefore the disease trajectories identified represent only severe cases. Furthermore, due to increased medical surveillance for breast cancer patients, these women may have been diagnosed with a disease that would continue to be undiagnosed in matched healthy individuals. Despite this, those diseases with an extremely high HR or with a long-term increased risk could not be solely explained by this possible surveillance bias. We have also endeavored to minimize this influence by starting the follow-up time from 2 months after cancer diagnosis. Finally, tumor characteristics and treatment data for both breast cancer and other diseases were not available, and consequently, we were not able to study the specific effects of disease treatments. Further studies are also needed to test and validate the identified disease trajectories in other settings and populations.

## Conclusion

This study describes a comprehensive picture of disease incidence and mortality after breast cancer diagnosis. Despite the increased incidence of many diseases, the most prominent increased mortality risk for breast cancer patients was other solid cancers. Our study reaffirms that preventing death due to breast cancer should be the priority to prolong life for breast cancer patients, but closer surveillance of other solid cancers and multidisciplinary post-cancer care for treatment-related side effects is also needed.

## Additional files

Additional file 1: **Table S1.** Risk of other diseases after breast cancer diagnosis (based on all diagnoses), compared to matched individuals (*N* = 622,204). **Table S2.** Odds ratios for the significant disease trajectories after breast cancer diagnosis. **Table S3.** Risk of other diseases after breast cancer diagnosis (based on main diagnosis), compared to matched individuals (*N* = 622,204). **Figure S1.** Flow chart of the study population and analysis plan. **Figure S2.** Diagram of the steps for disease trajectory analysis. (DOCX 141 kb)

Additional file 2: Combined ICD-10 codes used for disease identification. (XLSX 61 kb)

## Data Availability

The registry-based datasets linked and analyzed in the current study are not publicly available due to Swedish law. The data are available by applying through the Swedish National Board of Health and Welfare and Statistics Sweden. Detailed information on data application can be found at https://bestalladata.socialstyrelsen.se/data-for-forskning/ and http://www.scb.se/Vara-tjanster/bestalla-mikrodata/
