# Supplementary material for: Disease trajectories and mortality among women diagnosed with breast cancer
Source: Breast Cancer Res. 2019 Aug 16;21:95. doi: 10.1186/s13058-019-1181-5 (PMC6698019; doi:10.1186/s13058-019-1181-5)
Supplement: Supplementary file 1 — Table S1. Risk of other diseases after breast cancer diagnosis (based on all diagnoses), compared to matched individuals (N = 622,204). Table S2. Odds ratios for the significant disease trajectories after breast cancer diagnosis. Table S3. Risk of other diseases after breast cancer diagnosis (based on main diagnosis), compared to matched individuals (N = 622,204). Figure S1. Flow chart of the study population and analysis plan. Figure S2. Diagram of the steps for disease trajectory analysis. (DOCX 141 kb) [file 13058_2019_1181_MOESM1_ESM.docx]

**Table S1.**  Risk of other diseases after breast cancer diagnosis (based on all diagnoses)**^a^**, compared to matched individuals (N=622,204)

| Code | Disease | No. of cases | HR (95%CI) | P-value |  |
| --- | --- | --- | --- | --- | --- |
| A00 | Bacterial intestinal infections | 445 | 1.68 (1.51-1.85) | 2.40E-23 | |
| A08 | Other specified intestinal infections | 216 | 1.18 (1.02-1.36) | 0.0253 | |
| A09 | Infectious gastroenteritis and colitis | 592 | 1.35 (1.23-1.47) | 1.90E-11 | |
| A41 | Sepsis | 1258 | 2.27 (2.14-2.42) | 2.00E-148 | |
| A46 | Erysipelas | 1733 | 3.71 (3.51-3.93) | 0 | |
| A49 | Other bacterial diseases excluding septicemia and erysipelas | 475 | 2.85 (2.57-3.16) | 1.20E-87 | |
| A60 | Viral STD | 136 | 1.64 (1.36-1.96) | 1.20E-07 | |
| A69 | Other spirochetal diseases | 204 | 1.29 (1.11-1.49) | 6.90E-04 | |
| B09 | Viral infections characterized by skin and mucous membrane lesions | 888 | 1.66 (1.54-1.78) | 4.50E-43 | |
| B34 | Other viral diseases | 605 | 1.88 (1.73-2.06) | 1.80E-45 | |
| B49 | Mycoses | 1948 | 2.35 (2.24-2.47) | 2.00E-246 | |
| B96 | Bacterial infectious agents | 1030 | 1.72 (1.61-1.84) | 5.30E-57 | |
| B99 | Other infectious diseases | 882 | 3.60 (3.33-3.89) | 1.00E-225 | |
| C00 | Solid malignancies other than breast cancer | 2012 | 1.23 (1.17-1.29) | 8.40E-18 | |
| C81 | Hematological and lymphoid malignancies | 195 | 0.98 (0.85-1.14) | 0.8235 | |
| D00 | Other cancer in situ | 641 | 1.20 (1.11-1.31) | 1.20E-05 | |
| D50 | Anemia | 3104 | 1.70 (1.64-1.77) | 6.00E-159 | |
| D68 | Secondary bleeding disorders | 204 | 1.56 (1.34-1.81) | 5.60E-09 | |
| D69 | Purpura and other hemorrhagic conditions | 373 | 2.39 (2.14-2.68) | 1.60E-50 | |
| D70 | Neutropenia | 2325 | 20.0 (18.6-21.5) | 0 | |
| D72 | Eosinophilia | 110 | 4.50 (3.58-5.66) | 3.80E-38 | |
| E00 | Hypothyroid conditions | 1658 | 1.22 (1.16-1.29) | 3.20E-14 | |
| E02 | Thyroid disorders | 193 | 1.23 (1.06-1.43) | 0.0062 | |
| E04 | Non-toxic goiter | 488 | 1.30 (1.18-1.43) | 6.30E-08 | |
| E05 | Graves’ disease | 470 | 1.22 (1.11-1.34) | 5.80E-05 | |
| E10 | Diabetes | 2017 | 1.16 (1.11-1.22) | 4.10E-10 | |
| E21 | Hyperparathyroidism | 264 | 1.31 (1.15-1.49) | 4.90E-05 | |
| E40 | Malnutrition | 188 | 0.98 (0.85-1.14) | 0.8287 | |
| E65 | Obesity and other hypernutrition disorders | 693 | 1.04 (0.96-1.13) | 0.3137 | |
| E70 | Other Metabolic disorders | 1706 | 0.84 (0.80-0.89) | 1.80E-11 | |
| E83 | Disorders of mineral metabolism | 364 | 2.85 (2.53-3.21) | 2.30E-67 | |
| E86 | Volume depletion | 1373 | 1.46 (1.37-1.54) | 1.30E-37 | |
| E89 | Oth postproc endocrine and metabolic comp and disorders | 200 | 1.49 (1.29-1.73) | 1.40E-07 | |
| F00 | Dementia | 817 | 0.97 (0.91-1.05) | 0.4933 | |
| F05 | Delirium due to known physiological condition | 110 | 1.31 (1.07-1.60) | 0.0081 | |
| F06 | Oth mental disorders due to known physiological condition | 456 | 1.13 (1.03-1.25) | 0.0113 | |
| F10 | Substances abuse | 720 | 1.14 (1.06-1.23) | 9.20E-04 | |
| F20 | Schizophrenia | 147 | 0.98 (0.83-1.17) | 0.8604 | |
| F30 | Bipolar disorder | 191 | 1.13 (0.97-1.32) | 0.1065 | |
| F32 | Depression | 1835 | 1.55 (1.47-1.63) | 1.80E-66 | |
| F39 | Other mood disorder | 155 | 1.26 (1.06-1.49) | 0.0076 | |
| F40 | Anxiety | 1502 | 1.70 (1.61-1.80) | 2.20E-79 | |
| F43 | Reaction to severe stress | 800 | 1.71 (1.58-1.84) | 8.60E-44 | |
| F45 | Somatoform disorder | 241 | 1.20 (1.05-1.37) | 0.0078 | |
| F51 | Sleep disorder not due to a sub or known physiological condition | 144 | 1.67 (1.40-1.99) | 1.50E-08 | |
| G00 | Meningitis, encephalitis and abscess in the CNS | 103 | 1.26 (1.02-1.55) | 0.0288 | |
| G20 | Diseases of the basal ganglia and movement disorders | 506 | 1.21 (1.10-1.32) | 8.20E-05 | |
| G30 | Other degenerative diseases in the CNS (including Alzheimer) | 441 | 1.00 (0.91-1.11) | 0.9339 | |
| G40 | Epilepsia | 364 | 1.33 (1.19-1.48) | 5.00E-07 | |
| G43 | Migraine | 375 | 0.98 (0.88-1.09) | 0.7004 | |
| G44 | Other headache syndromes | 269 | 1.01 (0.89-1.15) | 0.8615 | |
| G45 | Transient cerebral ischemic attack | 715 | 0.97 (0.90-1.05) | 0.4555 | |
| G47 | Sleep disorder | 652 | 1.00 (0.92-1.08) | 0.9811 | |
| G50 | Diseases in nerves, nerve roots and nerve plexa | 1936 | 1.30 (1.24-1.36) | 2.40E-26 | |
| G60 | Polyneuropathies | 368 | 1.79 (1.60-2.00) | 3.00E-24 | |
| G80 | Cerebral palsy and other paralytic syndromes | 314 | 1.30 (1.15-1.46) | 1.50E-05 | |
| H00 | Hordeolum (stye) and chalazion | 256 | 1.30 (1.14-1.48) | 9.50E-05 | |
| H01 | Inflammation of eyelid | 575 | 1.18 (1.08-1.29) | 1.70E-04 | |
| H02 | Disorder of eyelid | 799 | 1.09 (1.02-1.18) | 0.0172 | |
| H04 | Disorder of lacrimal system | 1679 | 1.24 (1.18-1.31) | 5.20E-17 | |
| H10 | Diseases of the conjunctivae | 1256 | 1.15 (1.09-1.22) | 2.90E-06 | |
| H15 | Diseases of the sclerae, corneae, iridae and corpus ciliaris | 1351 | 1.09 (1.03-1.15) | 0.0032 | |
| H25 | Disorders of the lens | 6926 | 1.17 (1.14-1.20) | 2.40E-33 | |
| H33 | Other retinal detachments | 370 | 1.17 (1.05-1.30) | 0.0055 | |
| H34 | Retinal vascular occlusion | 285 | 0.98 (0.87-1.11) | 0.7698 | |
| H35 | Retinal disorders | 2677 | 1.18 (1.13-1.23) | 2.40E-15 | |
| H36 | Retinal disorders in diseases classified elsewhere | 385 | 0.97 (0.88-1.08) | 0.6114 | |
| H40 | Glaucoma | 1862 | 1.01 (0.96-1.06) | 0.6907 | |
| H43 | Disorders of the vitreous body and globe | 2752 | 1.12 (1.07-1.16) | 4.40E-08 | |
| H46 | Disorders of the optic nerve and visual pathways | 124 | 1.34 (1.11-1.62) | 0.0024 | |
| H49 | Disorders of the ocular muscles, binocular movement, and refraction | 1017 | 1.22 (1.15-1.31) | 1.80E-09 | |
| H53 | Visual disturbances and blindness | 1013 | 1.23 (1.15-1.31) | 8.80E-10 | |
| H59 | Other disorders of eye and adnexa | 187 | 1.05 (0.91-1.23) | 0.4923 | |
| H60 | Diseases of the external ear | 880 | 1.11 (1.03-1.19) | 0.0049 | |
| H65 | Diseases of the middle ear and mastoid | 290 | 1.19 (1.06-1.35) | 0.0048 | |
| H80 | Diseases of the inner ear | 1281 | 1.09 (1.02-1.15) | 0.0059 | |
| H90 | Other disorders of the ear | 2279 | 1.13 (1.08-1.18) | 9.20E-08 | |
| I10 | Hypertensive disorders | 6345 | 1.03 (1.00-1.05) | 0.0507 | |
| I20 | Angina pectoris | 1051 | 0.85 (0.80-0.90) | 3.90E-07 | |
| I21 | Ischemic heart disease | 1440 | 0.96 (0.91-1.01) | 0.1326 | |
| I26 | Venous thromboembolism | 3035 | 2.99 (2.86-3.11) | 0 | |
| I27 | Other pulmonary heart diseases | 137 | 1.25 (1.04-1.49) | 0.0164 | |
| I30 | Diseases of pericardium | 200 | 3.16 (2.69-3.71) | 1.40E-44 | |
| I34 | Non-rheumatic valve disorders | 621 | 1.00 (0.92-1.09) | 0.9811 | |
| I42 | Cardiomyopathy | 230 | 2.04 (1.76-2.35) | 2.40E-22 | |
| I49 | Cardiac arrhythmia | 2736 | 1.13 (1.08-1.17) | 4.20E-09 | |
| I50 | Heart failure | 1905 | 1.40 (1.34-1.47) | 4.40E-42 | |
| I51 | Complications and ill-defined descriptions of heart disease | 125 | 1.52 (1.26-1.84) | 1.50E-05 | |
| I60 | Stroke | 1525 | 1.01 (0.96-1.07) | 0.7122 | |
| I69 | Other sequelae following cerebrovascular disease | 777 | 1.00 (0.93-1.07) | 0.9305 | |
| I70 | Chronic total occlusion of artery of the extremities | 409 | 0.95 (0.85-1.05) | 0.2969 | |
| I71 | aneurysm and dissection | 228 | 1.08 (0.94-1.24) | 0.2737 | |
| I73 | Peripheral vascular disease | 238 | 0.84 (0.73-0.95) | 0.0082 | |
| I74 | Arterial embolism and thrombosis | 239 | 2.22 (1.92-2.56) | 6.00E-28 | |
| I77 | Disorder of arteries capillaries, and arterioles | 314 | 1.29 (1.14-1.45) | 2.70E-05 | |
| I83 | Varicose veins of lower extremities | 888 | 1.03 (0.96-1.11) | 0.3441 | |
| I84 | Haemorrhoids | 1095 | 1.30 (1.22-1.39) | 4.20E-16 | |
| I87 | Disorder of vein | 191 | 1.32 (1.14-1.54) | 2.90E-04 | |
| I89 | Other non-infectious diseases of the lymph vessels and lymph nodes, | 304 | 6.63 (5.72-7.69) | 2.00E-139 | |
| I95 | Hypotension | 419 | 1.26 (1.14-1.40) | 1.10E-05 | |
| I97 | Lymphedema | 928 | 56.4 (47.6-66.8) | 0 | |
| J00 | Upper respiratory infections and infections of the ear | 2395 | 1.46 (1.40-1.53) | 2.80E-66 | |
| J09 | Influenza | 128 | 1.46 (1.21-1.76) | 8.10E-05 | |
| J12 | Pneumonia | 2888 | 1.70 (1.63-1.77) | 8.00E-149 | |
| J20 | Other Lower respiratory infections | 645 | 1.42 (1.31-1.54) | 1.70E-16 | |
| J30 | Rhinitis | 876 | 1.07 (1.00-1.15) | 0.0546 | |
| J33 | Nasal polyp | 126 | 1.06 (0.88-1.28) | 0.5107 | |
| J34 | Disorder of nose and nasal sinuses | 281 | 1.17 (1.04-1.33) | 0.0116 | |
| J38 | Diseases of the vocal folds and larynx, not elsewhere specified | 411 | 1.86 (1.67-2.07) | 2.90E-30 | |
| J41 | Chronic obstructive pulmonary disease | 1281 | 1.08 (1.02-1.15) | 0.0082 | |
| J45 | Asthma | 968 | 1.00 (0.93-1.07) | 0.9802 | |
| J70 | Respiratory conditions due to external causes | 132 | 12.9 (9.89-16.8) | 2.90E-80 | |
| J82 | Pulmonary eosinophilia, not elsewhere classified | 185 | 1.94 (1.65-2.27) | 4.30E-16 | |
| J90 | Pleural effusion | 1273 | 4.30 (4.02-4.60) | 0 | |
| J93 | Pneumothorax | 189 | 3.22 (2.72-3.80) | 3.90E-43 | |
| J96 | Respiratory failure with hypercapnia | 593 | 1.39 (1.28-1.52) | 6.20E-14 | |
| J98 | Respiratory disorder | 162 | 1.63 (1.38-1.93) | 1.10E-08 | |
| K00 | Disease of hard tissue of teeth | 154 | 2.05 (1.72-2.44) | 1.10E-15 | |
| K04 | Diseases of pulp and periapical tissues | 208 | 1.70 (1.46-1.97) | 2.40E-12 | |
| K07 | Dentofacial anomalies | 173 | 1.03 (0.88-1.21) | 0.6899 | |
| K08 | Disorder of teeth and supporting structures | 123 | 1.02 (0.85-1.24) | 0.7984 | |
| K11 | Disease of salivary gland | 286 | 1.34 (1.18-1.52) | 3.50E-06 | |
| K12 | Stomatitis and related lesions | 184 | 2.04 (1.74-2.40) | 2.50E-18 | |
| K13 | Other lesions of oral mucosa | 202 | 1.43 (1.23-1.66) | 2.30E-06 | |
| K14 | Disease of tongue | 109 | 1.42 (1.16-1.74) | 6.80E-04 | |
| K20 | Esophagitis | 298 | 1.26 (1.11-1.42) | 2.40E-04 | |
| K21 | Gastro-esophageal reflux disease without esophagitis | 1043 | 1.12 (1.05-1.19) | 7.30E-04 | |
| K22 | Disease of esophagus | 264 | 1.31 (1.15-1.49) | 4.10E-05 | |
| K25 | Gastric ulcer, unsp as acute or chronic, w/o hemor or perf | 443 | 1.09 (0.99-1.21) | 0.0814 | |
| K26 | Duodenal ulcer, unsp as acute or chronic, w/o hemor or perf | 254 | 1.25 (1.10-1.43) | 8.80E-04 | |
| K29 | Gastroduodenitis, with bleeding | 1098 | 1.14 (1.07-1.21) | 4.70E-05 | |
| K30 | Functional dyspepsia | 602 | 1.15 (1.06-1.26) | 9.50E-04 | |
| K31 | Disease of stomach and duodenum | 180 | 1.17 (1.00-1.36) | 0.0507 | |
| K35 | Appendicitis | 270 | 1.23 (1.08-1.39) | 0.0017 | |
| K40 | Hernia | 1762 | 1.13 (1.08-1.19) | 8.20E-07 | |
| K50 | Inflammatory bowel disease | 252 | 1.36 (1.19-1.56) | 4.80E-06 | |
| K52 | Noninfective gastroenteritis and colitis | 604 | 1.41 (1.29-1.54) | 5.30E-15 | |
| K56 | Ileus | 538 | 1.33 (1.22-1.46) | 4.60E-10 | |
| K57 | Diverticle of the intestine | 1485 | 1.16 (1.09-1.22) | 1.90E-07 | |
| K58 | Irritable bowel syndrome without diarrhea | 339 | 1.01 (0.90-1.13) | 0.8292 | |
| K59 | Functional intestinal disorder | 1742 | 1.57 (1.49-1.65) | 1.80E-66 | |
| K60 | Fissure and fistula of the anal and rectal regions | 296 | 1.84 (1.63-2.09) | 1.10E-21 | |
| K62 | Disease of anus and rectum | 745 | 1.29 (1.20-1.40) | 5.60E-11 | |
| K63 | Disease of intestines | 377 | 1.26 (1.13-1.40) | 3.60E-05 | |
| K65 | Peritonitis | 132 | 1.31 (1.09-1.57) | 0.0041 | |
| K72 | Hepatic failure, not elsewhere specified | 172 | 3.32 (2.79-3.96) | 1.50E-41 | |
| K76 | Liver disease | 187 | 1.53 (1.31-1.79) | 8.20E-08 | |
| K80 | Other cholelithiasis with obstruction | 1249 | 1.13 (1.07-1.20) | 3.50E-05 | |
| K81 | Cholecystitis | 208 | 1.32 (1.14-1.53) | 1.90E-04 | |
| K82 | Disease of gallbladder and biliary tract | 213 | 1.31 (1.14-1.52) | 2.20E-04 | |
| K85 | Acute pancreatitis with infected necrosis | 224 | 1.23 (1.07-1.41) | 0.0038 | |
| K92 | Other disease of digestive system | 866 | 1.31 (1.22-1.40) | 2.50E-13 | |
| L01 | Other skin and subcutaneous infections | 1787 | 2.53 (2.40-2.66) | 2.00E-261 | |
| L20 | Dermatitis | 2773 | 1.41 (1.35-1.47) | 9.50E-63 | |
| L40 | Papulosquamous disorders | 925 | 1.12 (1.04-1.20) | 0.0015 | |
| L50 | Urticaria | 477 | 1.35 (1.22-1.48) | 1.30E-09 | |
| L51 | Erythema | 238 | 2.52 (2.18-2.90) | 1.60E-36 | |
| L57 | Skin changes due to chronic exposure to non-ionizing radiation | 1994 | 1.13 (1.08-1.18) | 3.70E-07 | |
| L58 | Radiodermatitis | 550 | 60.9 (48.6-76.3) | 3.00E-280 | |
| L60 | Nail disorders | 247 | 2.03 (1.77-2.33) | 7.10E-24 | |
| L63 | Alopecia | 173 | 1.24 (1.06-1.45) | 0.0086 | |
| L71 | Rosacea | 414 | 1.20 (1.08-1.33) | 5.70E-04 | |
| L72 | Follicular cyst of the skin and subcutaneous tissue | 656 | 1.64 (1.51-1.79) | 1.30E-31 | |
| L73 | Follicular disorders | 222 | 1.50 (1.30-1.73) | 1.70E-08 | |
| L81 | Disorder of pigmentation | 633 | 1.24 (1.14-1.35) | 5.00E-07 | |
| L82 | Other seborrheic keratosis | 2155 | 1.33 (1.27-1.39) | 5.60E-35 | |
| L85 | Epidermal thickening | 212 | 1.12 (0.97-1.30) | 0.1099 | |
| L89 | Pressure ulcer | 195 | 1.34 (1.15-1.55) | 1.90E-04 | |
| L90 | Atrophic skin diseases | 1043 | 1.93 (1.80-2.06) | 3.20E-82 | |
| L91 | Hypertrophic skin diseases | 276 | 2.65 (2.32-3.03) | 2.70E-46 | |
| L92 | Granulomatous disorder of the skin | 112 | 1.23 (1.01-1.50) | 0.0364 | |
| L97 | Non-prs chronic ulc unsp prt of l low leg w unsp severity | 246 | 0.97 (0.85-1.11) | 0.6286 | |
| L98 | Disorder of the skin and subcutaneous tissue | 611 | 1.62 (1.49-1.77) | 4.00E-28 | |
| M00 | Infectious arthropathies | 277 | 1.22 (1.08-1.39) | 0.0016 | |
| M04 | Autoimmune arthritis | 489 | 0.92 (0.84-1.01) | 0.0937 | |
| M14 | Other Inflammatory polyarthropathies | 574 | 1.09 (1.00-1.19) | 0.06 | |
| M15 | Osteoarthritis | 4092 | 1.07 (1.04-1.11) | 2.80E-05 | |
| M20 | Other joint disorders | 3371 | 1.10 (1.06-1.14) | 1.10E-07 | |
| M30 | Systemic connective tissue disorders | 616 | 0.89 (0.82-0.96) | 0.0044 | |
| M40 | Deforming dorsopathies | 271 | 1.16 (1.02-1.32) | 0.0215 | |
| M46 | Unsp inflammatory spondylopathy, multiple sites in spine | 105 | 0.98 (0.80-1.20) | 0.8546 | |
| M47 | Spondylosis | 326 | 1.11 (0.99-1.24) | 0.0833 | |
| M48 | Spondylopathy | 1192 | 1.15 (1.09-1.23) | 3.70E-06 | |
| M50 | Cervical disc disorder, cervicothoracic region | 469 | 1.03 (0.94-1.13) | 0.5355 | |
| M53 | Dorsopathy | 217 | 1.00 (0.87-1.15) | 0.9896 | |
| M54 | Dorsalgia | 2801 | 1.34 (1.28-1.39) | 1.00E-45 | |
| M60 | Disorders of muscles | 246 | 1.21 (1.06-1.38) | 0.0057 | |
| M65 | Disorders of synovium and tendons | 1562 | 1.26 (1.19-1.33) | 2.40E-17 | |
| M70 | Unspecified soft tissue disorders related to use/pressure | 652 | 1.19 (1.09-1.29) | 4.50E-05 | |
| M71 | Bursopathy | 159 | 1.00 (0.85-1.18) | 0.9812 | |
| M72 | Fibroblastic disorder | 259 | 1.07 (0.94-1.21) | 0.3294 | |
| M75 | Shoulder lesion | 923 | 1.08 (1.01-1.15) | 0.0326 | |
| M77 | Enthesopathy | 783 | 1.13 (1.05-1.22) | 9.50E-04 | |
| M79 | Myalgia and Pain in limb | 4425 | 1.29 (1.25-1.33) | 3.90E-56 | |
| M80 | Osteoporosis | 2255 | 1.94 (1.85-2.03) | 3.00E-176 | |
| M84 | Disorder of continuity of bone | 297 | 1.31 (1.16-1.48) | 1.60E-05 | |
| M85 | Other Disorders of bone density and structure | 927 | 5.04 (4.65-5.46) | 0 | |
| M87 | Osteonecrosis | 148 | 1.24 (1.05-1.48) | 0.0127 | |
| M90 | Osteopathy | 372 | 10.4 (8.99-12.1) | 2.00E-209 | |
| M93 | Chondropathies | 111 | 1.06 (0.87-1.30) | 0.5312 | |
| N13 | Obstructive and reflux uropathy | 317 | 1.91 (1.69-2.16) | 2.40E-25 | |
| N17 | Renal failure | 700 | 1.16 (1.08-1.26) | 1.70E-04 | |
| N20 | Urolithiasis | 422 | 1.02 (0.92-1.12) | 0.7547 | |
| N31 | Neuromuscular dysfunction of bladder | 156 | 1.20 (1.02-1.42) | 0.029 | |
| N39 | Disorders of urinary system, possibly infection | 4835 | 1.41 (1.37-1.45) | 6.00E-106 | |
| N60 | Benign mammary dysplasia | 350 | 1.55 (1.39-1.74) | 2.70E-14 | |
| N61 | Inflammatory disorders of breast | 393 | 9.67 (8.40-11.1) | 6.00E-220 | |
| N62 | Hypertrophy of breast | 1593 | 20.6 (18.8-22.4) | 0 | |
| N63 | Lump in breast | 1611 | 5.43 (5.11-5.78) | 0 | |
| N64 | Other disorders of breast | 1064 | 3.97 (3.69-4.26) | 0 | |
| N70 | Inflammatory diseases of the female pelvic organs | 1787 | 1.73 (1.64-1.82) | 2.10E-98 | |
| N80 | Endometriosis | 191 | 1.58 (1.36-1.84) | 5.20E-09 | |
| N81 | Female genital prolapse | 1713 | 1.15 (1.10-1.21) | 2.60E-08 | |
| N83 | Non-inflammatory ovarian diseases, fallopian tubes & broad ligaments | 1635 | 2.23 (2.11-2.36) | 9.00E-185 | |
| N84 | Polyp of female genital tract | 1710 | 2.84 (2.69-3.00) | 0 | |
| N85 | Other non-inflammatory diseases of the uterus, except cervix | 362 | 3.56 (3.15-4.02) | 1.00E-93 | |
| N87 | Dysplasia of cervix uteri | 410 | 1.34 (1.20-1.48) | 4.10E-08 | |
| N88 | Non-inflammatory disorders of cervix uteri | 192 | 1.40 (1.20-1.63) | 1.70E-05 | |
| N89 | Non-inflammatory vaginal diseases | 405 | 1.88 (1.69-2.09) | 7.50E-31 | |
| N90 | Non-inflammatory disorders of vulva and perineum | 278 | 1.19 (1.05-1.34) | 0.0074 | |
| N92 | Irregular menstruation | 1227 | 1.37 (1.29-1.46) | 1.30E-24 | |
| N93 | Other abnormal uterine and vaginal bleeding | 467 | 1.85 (1.68-2.05) | 5.00E-34 | |
| N94 | Unsp cond assoc w female genital organs and menstrual cycle | 386 | 1.19 (1.07-1.32) | 0.0016 | |
| N95 | Menopausal and perimenopausal disorders | 6121 | 1.56 (1.52-1.61) | 8.00E-223 | |
| N99 | Oth postprocedural complications and disorders of GU sys | 124 | 1.14 (0.94-1.37) | 0.1844 | |

Hazard ratios of various diseases among a Swedish national cohort of breast cancer patients, compared to women from the general population (matched on year of birth, county of residence and social economic status).

^a^Disease code here is a combined code from Swedish ICD code. Code transferring file can be found in Additional file 2.

**Table S2**. Odds ratios for the significant trajectories after breast cancer diagnosis

| Directional disease pairs | No. of pairs | OR (95%CI) |
| --- | --- | --- |
| C00→K56 | 83 | 14.8 (9.50-23.0) |
| D70→F32 | 69 | 1.72 (1.29-2.27) |
| D70→F40 | 75 | 2.15 (1.61-2.85) |
| D70→L20 | 83 | 1.67 (1.29-2.16) |
| D70→M54 | 75 | 1.73 (1.32-2.26) |
| D70→M80 | 80 | 1.89 (1.45-2.46) |
| H25→B96 | 100 | 1.77 (1.35-2.33) |
| M15→D50 | 167 | 1.62 (1.35-1.96) |
| M15→J96 | 57 | 2.80 (1.97-4.00) |
| M20→H25 | 261 | 1.29 (1.12-1.49) |
| M54→M48 | 190 | 8.80 (6.86-11.3) |
| N83→C00 | 58 | 1.96 (1.44-2.68) |
| N95→C00 | 207 | 1.81 (1.54-2.14) |
| N95→H04 | 121 | 1.47 (1.18-1.83) |
| N95→N84 | 374 | 6.36 (5.37-7.53) |

**Table S3.** Risk of other diseases after breast cancer diagnosis (based on main diagnosis), compared to matched individuals (N=622,204)

| Code | Name of disease | No. of cases | | HR (95% CI) | |
| --- | --- | --- | --- | --- | --- |
| A00 | Bacterial intestinal infections | | 288 | 1.58 (1.39-1.79) |  |
| A09 | Infectious gastroenteritis and colitis | | 517 | 1.29 (1.18-1.42) |  |
| A41 | Sepsis | | 886 | 2.22 (2.06-2.39) |  |
| A46 | Erysipelas | | 1552 | 3.56 (3.35-3.77) |  |
| A49 | Other bacterial diseases excluding septicemia and erysipelas | | 283 | 2.53 (2.21-2.88) |  |
| A60 | Viral STD | | 117 | 1.56 (1.28-1.90) |  |
| B09 | Viral infections characterized by skin and mucous membrane lesions | | 710 | 1.61 (1.49-1.75) |  |
| B34 | Other viral diseases | | 521 | 1.90 (1.72-2.08) |  |
| B49 | Mycoses | | 1064 | 2.21 (2.07-2.37) |  |
| B96 | Bacterial infectious agents | | 18 | 1.60 (0.97-2.63) |  |
| B99 | Other infectious disease | | 604 | 3.46 (3.15-3.80) |  |
| D00 | Other cancer in situ | | 465 | 1.38 (1.25-1.52) |  |
| D25 | Benign gynecological tumor | | 1481 | 1.62 (1.53-1.71) |  |
| D48 | Uncertain tumor | | 5409 | 2.14 (2.08-2.21) |  |
| D50 | Anemia | | 1013 | 1.31 (1.22-1.40) |  |
| D68 | Secondary bleeding disorders | | 76 | 1.74 (1.36-2.22) |  |
| D69 | Hemorrhagic condition | | 167 | 1.92 (1.62-2.27) |  |
| D70 | Neutropenia | | 1128 | 25.0 (22.4-27.9) |  |
| D72 | Eosinophilia | | 35 | 2.28 (1.57-3.30) |  |
| E00 | Hypothyroid conditions | | 265 | 1.20 (1.05-1.36) |  |
| E04 | Nontoxic goiter | | 480 | 1.21 (1.10-1.33) |  |
| E05 | Graves’ disease | | 503 | 1.19 (1.09-1.31) |  |
| E10 | Diabetes | | 1493 | 1.10 (1.04-1.16) |  |
| E21 | Hyperparathyroidism | | 259 | 1.27 (1.11-1.44) |  |
| E70 | Other Metabolic disorders | | 169 | 0.87 (0.74-1.02) |  |
| E83 | Disorder of mineral metabolism | | 175 | 2.33 (1.97-2.75) |  |
| E86 | Volume depletion | | 441 | 1.31 (1.18-1.45) |  |
| E89 | Other postproc endocrine and metabolic comp and disorders | | 143 | 1.53 (1.28-1.83) |  |
| F32 | Depression | | 1519 | 1.46 (1.39-1.55) |  |
| F40 | Anxiety | | 1284 | 1.63 (1.54-1.73) |  |
| F43 | Reaction to severe stress | | 732 | 1.72 (1.59-1.86) |  |
| F51 | Sleep disorder not due to a sub or known physiological condition | | 90 | 1.72 (1.37-2.15) |  |
| G20 | Diseases of the basal ganglia and movement disorders | | 528 | 1.11 (1.01-1.21) |  |
| G40 | Epilepsia | | 349 | 1.06 (0.95-1.19) |  |
| G50 | Diseases in nerves, nerve roots and nerve plexa | | 1858 | 1.23 (1.17-1.29) |  |
| G60 | Polyneuropathies | | 264 | 1.75 (1.53-1.99) |  |
| G80 | Cerebral palsy and other paralytic syndromes | | 96 | 1.19 (0.96-1.47) |  |
| H00 | Chalazion eye eyelid | | 240 | 1.34 (1.17-1.53) |  |
| H01 | inflammation of eyelid | | 476 | 1.22 (1.11-1.34) |  |
| H04 | Disorder of lacrimal system | | 1360 | 1.23 (1.16-1.30) |  |
| H10 | Diseases of the conjunctivae | | 1096 | 1.12 (1.05-1.19) |  |
| H25 | Disorders of the lens | | 7150 | 1.14 (1.11-1.17) |  |
| H35 | Retinal disorder | | 2028 | 1.16 (1.11-1.22) |  |
| H43 | Disorders of the vitreous body and globe | | 2585 | 1.11 (1.06-1.16) |  |
| H49 | Disorders of the ocular muscles, binocular movement and refraction | | 669 | 1.23 (1.13-1.33) |  |
| H53 | Visual disturbances and blindness | | 686 | 1.25 (1.15-1.35) |  |
| H90 | Other disorders of the ear | | 2264 | 1.11 (1.07-1.16) |  |
| I20 | Angina pectoris | | 956 | 0.81 (0.76-0.86) |  |
| I26 | Venous thromboembolism | | 2754 | 2.89 (2.76-3.01) |  |
| I30 | Diseases of pericardium | | 134 | 3.31 (2.72-4.04) |  |
| I42 | Cardiomyopathy | | 188 | 1.69 (1.44-1.97) |  |
| I49 | Cardiac arrhythmia | | 2691 | 1.14 (1.09-1.19) |  |
| I50 | Heart failure | | 1185 | 1.38 (1.30-1.47) |  |
| I51 | Complications and ill-defined descriptions of heart disease | | 89 | 1.76 (1.40-2.21) |  |
| I74 | Embolism and thrombosis of artery | | 171 | 2.09 (1.77-2.47) |  |
| I77 | Disorder of arteries capillaries, and arterioles | | 203 | 1.30 (1.12-1.51) |  |
| I84 | Haemorrhoids | | 999 | 1.24 (1.16-1.33) |  |
| I89 | Other non-infectious diseases of the lymph vessels and lymph nodes, unspecified | | 186 | 5.38 (4.49-6.45) |  |
| I95 | Hypotension | | 237 | 1.27 (1.11-1.45) |  |
| I97 | Lymphedema | | 507 | 65.2 (51.1-83.2) |  |
| J00 | Upper respiratory infections and infections of the ear | | 2251 | 1.39 (1.33-1.45) |  |
| J09 | Influenza | | 106 | 1.43 (1.17-1.76) |  |
| J12 | Pneumonia | | 2393 | 1.63 (1.56-1.70) |  |
| J20 | Other Lower respiratory infections | | 544 | 1.42 (1.29-1.55) |  |
| J38 | Other diseases of larynx | | 391 | 1.84 (1.65-2.05) |  |
| J70 | Respiratory conditions due to external agent | | 92 | 14.4 (10.4-19.9) |  |
| J82 | Pulmonary eosinophilia, not elsewhere classified | | 163 | 1.83 (1.55-2.16) |  |
| J90 | Pleural effusion | | 689 | 5.03 (4.59-5.53) |  |
| J93 | Pneumothorax | | 96 | 3.06 (2.43-3.86) |  |
| J96 | Respiratory failure with hypercapnia | | 286 | 1.47 (1.29-1.66) |  |
| J98 | Respiratory disorders | | 133 | 1.76 (1.46-2.12) |  |
| K00 | Disease of hard tissue of teeth | | 115 | 1.78 (1.46-2.17) |  |
| K04 | Diseases of pulp and periapical tissues | | 178 | 1.64 (1.40-1.92) |  |
| K11 | Disease of salivary gland | | 272 | 1.31 (1.15-1.49) |  |
| K12 | Stomatitis and related lesions | | 119 | 1.61 (1.33-1.96) |  |
| K13 | Other lesions of oral mucosa | | 174 | 1.35 (1.15-1.59) |  |
| K22 | Disease of esophagus | | 210 | 1.26 (1.09-1.45) |  |
| K29 | Gastroduodenitis, with bleeding | | 752 | 1.09 (1.01-1.18) |  |
| K40 | Hernia | | 1444 | 1.12 (1.06-1.18) |  |
| K50 | Inflammatory bowel disease | | 433 | 1.23 (1.11-1.36) |  |
| K52 | Non-infective gastroenteritis and colitis | | 551 | 1.33 (1.22-1.46) |  |
| K56 | Ileus | | 464 | 1.30 (1.18-1.43) |  |
| K57 | Diverticle of the intestine | | 1381 | 1.16 (1.10-1.23) |  |
| K59 | Functional intestinal disorder | | 1263 | 1.41 (1.33-1.50) |  |
| K60 | Fissure and fistula of the anal and rectal regions | | 271 | 1.71 (1.50-1.95) |  |
| K62 | Disease of anus and rectum | | 658 | 1.26 (1.16-1.37) |  |
| K63 | Disease of intestine | | 274 | 1.21 (1.07-1.38) |  |
| K72 | Hepatic failure with coma | | 70 | 2.99 (2.29-3.92) |  |
| K76 | Liver disease | | 136 | 1.50 (1.25-1.80) |  |
| K80 | Other cholelithiasis with obstruction | | 1261 | 1.12 (1.05-1.18) |  |
| K81 | Cholecystitis | | 176 | 1.32 (1.12-1.54) |  |
| K92 | Other disease of digestive system | | 700 | 1.26 (1.16-1.36) |  |
| L01 | Other skin and subcutaneous infections | | 1315 | 2.28 (2.14-2.42) |  |
| L20 | Dermatitis | | 2521 | 1.37 (1.32-1.43) |  |
| L50 | Urticaria | | 410 | 1.33 (1.19-1.47) |  |
| L51 | Erythema | | 202 | 2.46 (2.11-2.87) |  |
| L57 | Skin changes due to chr expsr to nonionizing radiation | | 2069 | 1.10 (1.05-1.15) |  |
| L58 | Radiodermatitis | | 110 | 25.7 (18.0-36.6) |  |
| L60 | Nail disorder | | 218 | 2.00 (1.73-2.32) |  |
| L72 | Follicular cyst of the skin and subcutaneous tissue | | 577 | 1.59 (1.46-1.74) |  |
| L73 | Follicular disorder | | 180 | 1.53 (1.30-1.79) |  |
| L81 | Disorder of pigmentation | | 421 | 1.28 (1.15-1.42) |  |
| L82 | Other seborrheic keratosis | | 1685 | 1.35 (1.28-1.42) |  |
| L89 | Pressure ulcer | | 96 | 1.15 (0.93-1.43) |  |
| L90 | Atrophic disorder of skin | | 882 | 1.61 (1.50-1.73) |  |
| L91 | Hypertrophic disorder of the skin | | 181 | 2.90 (2.46-3.43) |  |
| L98 | Disorder of the skin and subcutaneous tissue | | 528 | 1.55 (1.41-1.70) |  |
| M15 | Osteoarthritis | | 4857 | 1.05 (1.02-1.09) |  |
| M20 | Other joint disorders | | 3491 | 1.06 (1.02-1.10) |  |
| M48 | Spondylopathy | | 1036 | 1.09 (1.03-1.17) |  |
| M54 | Dorsalgia | | 2584 | 1.25 (1.20-1.30) |  |
| M65 | Disorders of synovium and tendons | | 1468 | 1.24 (1.17-1.31) |  |
| M70 | Unsp soft tissue disord related to use/pressure mult sites | | 544 | 1.21 (1.10-1.32) |  |
| M79 | Myalgia and Pain in limb | | 4246 | 1.27 (1.23-1.32) |  |
| M80 | Osteoporosis | | 1841 | 2.29 (2.18-2.41) |  |
| M84 | Disorder of continuity of bone | | 238 | 1.17 (1.02-1.34) |  |
| M85 | Other Disorder of bone density and structure | | 927 | 4.71 (4.35-5.10) |  |
| M90 | Osteopathy | | 51 | 22.6 (13.6-37.7) |  |
| N13 | Obstructive and reflux uropathy | | 207 | 1.78 (1.53-2.07) |  |
| N17 | Renal failure | | 429 | 1.12 (1.01-1.24) |  |
| N39 | Disorder of urinary system, possibly infection | | 3929 | 1.32 (1.27-1.36) |  |
| N60 | Benign mammary dysplasia | | 300 | 1.26 (1.12-1.43) |  |
| N61 | Inflammatory disorders of breast | | 327 | 7.93 (6.84-9.18) |  |
| N62 | Hypertrophy of breast | | 984 | 12.1 (11.1-13.4) |  |
| N63 | Lump in breast | | 1591 | 4.82 (4.54-5.12) |  |
| N64 | Other disorders of breast | | 896 | 3.35 (3.10-3.61) |  |
| N70 | Inflammatory diseases of the female pelvic organs | | 1121 | 1.49 (1.40-1.58) |  |
| N80 | Endometriosis | | 100 | 1.49 (1.21-1.84) |  |
| N81 | Female genital prolapse | | 1855 | 1.12 (1.06-1.17) |  |
| N83 | Non-inflammatory ovarian diseases, fallopian tubes & broad ligaments | | 1498 | 2.22 (2.10-2.35) |  |
| N84 | Polyp of female genital tract | | 1378 | 3.20 (3.01-3.41) |  |
| N85 | Non-inflammatory disorder of uterus | | 285 | 3.81 (3.32-4.38) |  |
| N87 | Dysplasia of cervix uteri | | 431 | 1.30 (1.18-1.44) |  |
| N88 | Non-inflammatory disorder of cervix uteri | | 118 | 1.96 (1.61-2.39) |  |
| N89 | Non-inflammatory disorder of vagina | | 345 | 2.00 (1.78-2.25) |  |
| N92 | Irregular menstruation | | 1244 | 1.30 (1.22-1.38) |  |
| N93 | Abnormal uterine and vaginal bleeding | | 427 | 1.84 (1.66-2.04) |  |
| N95 | Menopausal and perimenopausal disorder | | 6233 | 1.42 (1.39-1.46) |  |

Hazard ratios of various diseases among a Swedish national cohort of breast cancer patients, compared to women from the general population (matched on year of birth, county of residence and social economic status). The analyses were based on the main diagnosis of those diseases with statistical significant results in Table S1.

**Figure S1**. Flow chart of the study population and analysis plan

**Corresponding General Population**

Women in Sweden

from the 1990 national census

*N*=4,343,853

**National Breast Cancer cohort**

All breast cancer patients

from the 1990 national census and Swedish Cancer Register

*N*=67,209

Matched cohort on year of birth, county of residence and social economic status Age= 20-80

Follow up to Dec 31, 2012

*N*=564,703

Breast cancer patients

Age= 20-80

Follow up to Dec 31, 2012

*N* =57,501

Matching

Swedish Patient/ Cancer Register

Disease incidence analysis comparing breast cancer patients to matched women

Swedish Cause of Death Register

Disease mortality analysis comparing breast cancer patients to matched women

Disease trajectory analysis in breast cancer patients

**Figure S2**. Diagram of the steps for disease trajectory analysis

Disease trajectory analysis

**Step 1.** 927 disease pairs (D1→D2) with more than 50 cases of D2 after D1 diagnosis in breast cancer patients

**Step 2.** 33 disease pairs (D1→D2) with a significantly higher probability of D2 diagnosed after D1

**Method**: binomial test was used to test whether the probability of D2 being diagnosed after D1 was significantly larger than 50%

**Step 3.** 15 disease pairs (D1→D2) with a significantly increased risk of D2 after D1 (OR>1). (results in Figure 2)

**Method**: nested case-control design: use conditional logistic regression to calculate OR of D2 after D1.

**Among the 137 diseases with significantly increased risk after breast cancer, compared to matched individuals (results in Figure 1)**

**Step 4**. 4 disease pairs (D1→D2) have increased breast cancer or other cancer mortality after D2 (OR>1), with more than 20 patients diagnosed with D1 and thereafter D2, and consequently died of breast cancer or other cancer. (results in Figure 3)

**Method**: nested case-control design: use conditional logistic regression to calculate OR of breast cancer or other cancer mortality after D2.
